# Physical activity and sedentary behavior trajectories and their associations with quality of life, disability, and all-cause mortality

**DOI:** 10.1186/s11556-022-00291-3

**Published:** 2022-04-29

**Authors:** Aarón Salinas-Rodríguez, Betty Manrique-Espinoza, Rosa Palazuelos-González, Ana Rivera-Almaraz, Alejandra Jáuregui

**Affiliations:** 1 Center for Evaluation and Surveys Research, National Institute of Public Health, Av. Universidad #655. Colonia Santa María Ahuacatitlan ZC, 62100 Cuernavaca, Mor Mexico; 2 Center for Research in Nutrition and Health, National Institute of Public Health, Av. Universidad #655. Colonia Santa María Ahuacatitlan ZC, 62100 Cuernavaca, Mor Mexico

**Keywords:** Physical activity, Sedentary behavior, Quality of life, Disability, All-cause mortality, Growth mixture modelling

## Abstract

**Background:**

Physical activity (PA) and sedentary behavior (SB) are not stable conditions but change over time and among individuals, and both could have deleterious effects on health-related outcomes among older adults. This study aimed to identify the longitudinal trajectories of PA and SB and estimate their association with quality of life, disability, and all-cause mortality in a national sample of older Mexican adults.

**Methods:**

Data comes from three waves of the WHO Study on global AGEing and adult health (SAGE) in Mexico (2009, 2014, 2017). In total, 3209 older adults ages 50 and above were included. PA and SB were determined by using the Global Physical Activity Questionnaire (GPAQ). Disability was measured using the WHO Disability Assessment Schedule (WHODAS 2.0), quality of life using the WHOQOL (WHO Quality of Life) instrument, and all-cause mortality using a verbal autopsy. We used growth mixture modeling (GMM) to investigate the longitudinal trajectories of PA and SB. Three-level linear mixed effect models were used to estimate the associations of PA and SB with quality of life and disability and the Cox model for the association with all-cause mortality.

**Results:**

Three longitudinal trajectories of PA and SB were found: low-PA-decreasers, moderate-PA-decreasers, and high-PA-decreasers for PA; and low-maintainers, steep-decreasers, and steep-increasers for SB. Decreased quality of life, increased disability, and all-cause mortality were all consistently associated with worse PA and SB trajectories.

**Conclusions:**

Our results highlight the need for health policies and prevention strategies that promote PA and limit SB in middle-aged adults. Further studies should consider these activities/behaviors as exposures that vary throughout life and work to identify vulnerable groups of older adults for whom physical activation interventions and programs would be most impactful.

**Supplementary Information:**

The online version contains supplementary material available at 10.1186/s11556-022-00291-3.

## Background

The increase in life expectancy and decline in fertility rates have raised older adult (OA) population, particularly in low and middle-income countries (LMIC) [1]. A high proportion of OA suffer from non-communicable diseases and geriatric syndromes that could be prevented through modifiable factors like physical activity (PA). Among OA, PA reduces the risk of cardiovascular disease, breast cancer, bone fractures, falls, disability, cognitive impairment, dementia, and Alzheimer disease, and has been associated with a higher quality of life (QoL) and healthy aging [2].

In contrast to PA, physical inactivity is responsible for a loss of over 13 million disability-adjusted-life years, mainly through non-communicable diseases [3]. With a longer average lifespan, more OA are prone to decreased PA and increased sedentary behaviors (SB), including activities that may have deleterious health consequences such as watching TV, sitting, reading, eating, motorized transportation, etcetera.

A growing body of evidence suggests the relevance of PA and SB for QoL, disability, and all-cause mortality. A harmonized meta-analysis study including more than 1 million people, concluded that adults with high levels of PA (60–75 min of moderate PA a day) seem to eliminate the mortality associated with sitting time [4]. However, this study was not specific for OA, a population that has been reported to make light intensity PA, and this intensity only appears to reduce the risk of chronic diseases and mortality. Also, a higher risk has been reported for cardiometabolic, bone, muscular and mental/cognitive health among OA with more hours of SB per day [5]. The specific association between PA and QoL has been reported in several cross-sectional and longitudinal studies [6–8]. Also, a reduction in the sedentary activities have been associated with physical independence and greater QoL [5].

Despite extensive evidence that associates PA and SB with QoL, disability, and mortality among OA, most studies have focused on the analysis of categorized groups of participants with a certain level of PA or certain hours of sitting time as a measure of SB. Fewer studies have investigated the role of these predictors as changing and non-static behaviors that vary from person to person over time, which is reflected in longitudinal trajectories of both PA and SB. Sanchez- Sanchez et al. [9] and Laddu et al. [10] identified different trajectories of PA and their association with adverse health outcomes in OA. Regarding SB, one study analyzed the sitting time trajectories and their association with frailty in middle-aged women [11]. To the best of our knowledge, there are not specific studies analyzing SB trajectories and their association with mortality, disability, or QoL among the OA population.

Given that PA and SB change over an individual’s life course [12], this study aimed to identify longitudinal trajectories of PA and SB and estimate their association with QoL, disability, and all-cause mortality in a national sample of Mexican OA. The main hypothesis was there is no “typical” trajectory for PA and SB, but rather that trajectories are heterogeneous. We also hypothesized that poor PA and SB trajectories would be associated with lower QoL, highest disability burden, and elevated mortality rates.

## Methods

### Population and sample

Data comes from the three waves of the World Health Organization (WHO) Study on global AGEing and adult health (SAGE) in Mexico. SAGE is a multi-country, longitudinal study, based on nationally representative samples of individuals aged ≥50y. It has been conducted in six countries -- China, Ghana, India, Mexico, Russia, and South Africa – which each bring different geographic distributions, population sizes, income levels (low and medium), and phases in the epidemiological transition. To date, SAGE has three longitudinal measurements in Mexico, details of the study design have been published elsewhere [13]. Briefly, Wave 1 (baseline) was collected between July and September 2009, with a total sample of 2404 respondents ≥50y. Wave 2 data was collected between July and October 2014, with a refreshed sample of 618 new interviews (also ≥50) additional to the remained sample of Wave 1; and Wave 3 from August to November 2017 with 2318 participants (including 610 new interviews). In total, 3277 individuals were interviewed in the three waves. Given that the aim of this study was to identify longitudinal trajectories of the PA and SB, we included participants with at least two measurements. The final analytical sample had 3209 subjects. The response rate (the proportion of those initially invited, i.e., those with baseline measurement) was 76% (Fig. S1 in the Additional file, Appendix 1). We identified baseline differences between the final sample and excluded participants in several analytical variables -- the latter were older and had a higher prevalence of frailty and multimorbidity (*p* < 0.05).

### Sample for mortality data

For all-cause mortality data analysis, sample definition proceeded as follows. The individuals were included if they had two of three complete measurements (censored observations) or had measurements for waves 1 and 2, and their death occurred between waves 2 and 3. Additionally, and given that our cohort includes incorporating new individuals (with rolling admissions), older adults with measurements in waves 2 and 3 were considered with delayed entry. Table 1 shows the different settings and their sample size. Hence, this analysis included a total of 2564 older adults.

**Table 1:** Analytical sample for survival analysis

Wave 1 2009	Wave 2 2014	Wave 3 2017	n
X	X (*censored*)		1394
	X (*delayed entry*)	X (*censored*)	218
X	X	X (*censored*)	584
X	X	failure (*died between waves 2 and 3*)	368
			2564

### Definition of variables

#### Outcomes

##### Quality of life (QoL)

We assessed this variable using the WHOQOL (WHO Quality of Life) instrument. This eight-item questionnaire covers the following core domains (two items per domain): physical, psychological, social, and environmental. The eight items are summed for an overall score ranging from 0 to 100. The higher the score, the higher QoL [14].

##### Disability

We used the WHO Disability Assessment Schedule Version 2.0 (WHODAS 2.0), a cross-culturally validated, 12-item tool that measures limitations in activity and daily-life participation over the last month. WHODAS 2.0 covers six domains within the 12 items (two per domain): 1) cognition and communication, 2) self-care, 3) mobility, 4) interpersonal relations, 5) life activities, and 6) participation. The results of the 12 items are summed to obtain a global score expressed on a continuous scale from 0 (no disability) to 100 (complete disability) [15].

##### All-cause mortality

Data drawn from the interviews in Wave 3 (2017) included information about death (for any cause) that occurred during the follow-up using the WHO-Verbal autopsy instrument, which ascertains and attributes causes of death based on the self-report of the closest relative -or non-relative person- of the older adults [16]. We define the follow-up time as the interval between the interviews of the baseline measurement (whether were individuals whose risk began in 2009 or with delayed entry in 2014) and the 3rd Wave for censored data. We recorded the date of death for the deceased, which provided information on the survival time. We then calculated the follow-up time according to the number of days elapsed.

#### Main exposures

##### Physical activity

This was assessed using the Global Physical Activity Questionnaire (GPAQ). The GPAQ measures PA in terms of intensity, duration, and frequency, and assesses three domains in which PA is performed: activity at work, travel to and from places, and recreational activities [17]. The total amount of moderate-to-vigorous PA in a typical week was calculated and METs/hours per week were reported [18].

##### Sedentary behavior

The GPAQ was also used to assess SB. In this instrument, participants were asked for the minutes per day spent in sitting activities [19, 20]. Specifically, OA reported the total time they usually spent sitting or reclining at work, at home, getting to and from places, or with friends (i.e., sitting at a desk, sitting with friends, travelling in car, bus, train, reading, playing cards, or watching television), excluding time spent sleeping. SB was used in the analysis as a continuous measurement of daily hours spent sitting.

#### Measurements

Height and weight -- measured through stadiometers and calibrated electronic weighting scales -- were used to calculate body mass index (kg/m2). Grip strength was measured twice for both hands using the hand dynamometer (Baseline Electronic Smedley Hand Dynamometer, Fabrication Enterprises, White Plains, NY, USA). Four-meter walk time was used to measure gait speed. Participants were asked to walk at a normal pace.

### Covariates

Covariates were categorized as follows: sex (1 = female), age, and number of years of formal education. Socioeconomic status (SES) of the household was derived using the WHO standard approach to estimate permanent income from household ownership of durable goods, dwelling characteristics (type of floors, walls, and cooking stove), and access to services such as water, sanitation, and electricity [21]. SES was included as a continuous variable, with higher values indicating better SES. Multimorbidity was included as a dichotomous variable (with/without multimorbidity) and was defined as the presence of two or more chronic non-communicable conditions from the list of nine chronic diseases included in the SAGE study. The operational definitions of these diseases have been published elsewhere [22]. Frailty status was determined using a modified frailty phenotype, based on the criteria proposed by Fried et al. [23], which includes five components: weight loss, exhaustion, low physical activity, slow walking speed and weakness. Details of the previous application of this frailty measurement in the SAGE sample has been published elsewhere [24]. We also used sarcopenia as an additional covariate. In accordance with previous publications, we defined the presence of sarcopenia as having low skeletal muscle mass (reflected by lower skeletal muscle mass index) and either a slow gait speed or weak handgrip strength. Details of the specific algorithms used to define sarcopenia status in the older adult population using the SAGE study sample are published elsewhere [25]. Finally, body mass index (BMI) was calculated using weight (kg) and height (cm) (BMI = Weight [kg] / Height [m2]) and was incorporated into the analysis as a continuous variable.

### Statistical analysis

Baseline characteristics are presented in percentages and means (standard deviation) as appropriate. Health and sociodemographic characteristics related to longitudinal trajectories of PA were compared using Chi-square or ANOVA tests.

We used growth mixture modelling (GMM) to investigate the longitudinal trajectories of PA and SB [26]. GMM is useful since it provides information regarding the growth factors of each different trajectory. The intercept and slope (growth factors) are interpreted as usual in longitudinal modelling: the level of outcome variable when time is equal to zero and the rate of change in the outcome over time, respectively. According to current recommendations [27], we initially specified a single-class latent growth curve model to determine the pattern of change over time. Given the number of available measurements in the SAGE study (i.e., three waves) we examined a linear and a quadratic pattern of change. We specifically applied the GMM with class-specific random intercepts. We also applied an exploratory approach and fitted models with an increasing number of classes to identify the optimal latent class model.

We determined the best model (i.e., the one with the optimal number of classes) based on statistical criteria, parsimony, and interpretability [27]. We considered: (1) the lowest values of the goodness of fit measures - Bayes Information Criteria (BIC), Akaike Information Criteria (AIC) and the sample-size adjusted BIC (aBIC), (2) the following versions of the likelihood ratio tests (LRT): Vuong-Lo-Mendell-Rubin, Lo-Mendell-Rubin adjusted, and Bootstrapped, (3) the way to which the trajectory classes captured distinct and important patterns in the data, and (4) the quality of the model in terms of posterior probability diagnostics, namely the entropy and average posterior probability for each trajectory class.

We analyzed the association between the PA and SB trajectories and the distal outcomes (QoL, disability, and all-cause mortality) using linear mixed-effects regression and Cox proportional hazards models. Given that the Mexico-SAGE study included multiple members from within the same household and had repeated measurements of QoL and disability from the same individual, our data had a three-level hierarchical structure, with measurement occasions at level 1, individuals at level 2, and households at level 3. We then fitted a random intercept models including the subject and household IDs as random effects for QoL and disability outcomes.

For the Cox model, new admissions are left-truncated observations (also known as delayed entry), implying that not all individuals start to be at risk simultaneously, which in turn represents a potential bias [28–30]. Then, we considered the delayed entry feature in our statistical analysis following the proposal of Lamarca et al. [31] to analyze left-truncated data with the older adult population using age as the time scale. In our study, consider the survival time as the elapsed time from age 50 until the event of interest. Additionally, we used clustered standard errors to account for correlation between repeated measurements within individuals.

All models were adjusted using the covariates described above. Furthermore, the modeling of PA was adjusted by weekly hours of SB, and SB was adjusted by PA (METs/hours per week), and both (PA and SB) for the follow-up time. Regression coefficients, hazard ratios, and 95% confidence intervals were reported.

Models for GMM were estimated in Mplus v8.5 by full maximum likelihood (FML) and robust standard errors to non-normality [32]. To avoid local maxima for the expectation-maximization (EM) algorithm, we estimated the models with 200 random starting values and 100 iterations per set of starting values. According to the guidelines for reporting on latent trajectory studies [27], the code syntax is provided in the Additional file, Appendix 2. We used Stata 17.0 to model the association between PA and SB trajectories and distal outcomes.

This study was conducted following the STROBE guidelines for reporting cohort studies (STROBE checklist is reported in the Additional file, Appendix 3).

## Results

At baseline, the sample was constituted of 2404 older adults. The mean age was 67.5 (st. dev. = 10.3), and 61.7% were female. The average PA (METs/hours per week) was 68.3 (st. dev. = 113.1), and the mean daily hours of SB was 2.6 (st. dev. = 2.6).

### Trajectories of PA and SB

The single-class latent growth curve model showed favorable evidence for the linear model (*p* < 0.01) over the quadratic model (*p* = 0.99) for both PA and SB. We adjusted models from one to four trajectories. Table 2 provides the information of the Model Selection Criteria for all the models tested. For PA and SB, the three-class model was the best according to the fit indices, entropy and the *p*-values associated with the three LRT used (Vuong-Lo-Mendell-Rubin, Lo-Mendell-Rubin adjusted, and Bootstrapped). Additionally, we selected the three-class model based on the lower values of AIC, BIC, and aBIC.

**Table 2:** Model Selection Criteria of the Growth Mixture Model (GMM) analysis

		Growth mixture model (GMM)							Likelihood Ratio Test *p*-value		
		Free parameters	LL	AIC	BIC	aBIC	Classes-size (%)	Entropy	Bootstrapped	Vuong-Lo-Mendell-Rubin	Lo-Mendell-Rubin
Physical activity	1-class	14	−23,840.52	47,709.05	47,791.9	47,747.4	100				
	2-classes	13	− 2758.87	5543.74	5620.63	5579.33	63,37	0.98	< 0.01	< 0.01	< 0.01
	3-classes	21	− 1682.76	3407.52	3531.72	3464.99	37,20,43	0.99	< 0.01	1.00	1.00
	4-classes	29	− 1909.13	3876.26	4047.78	3955.64	0,59,28,13	0.99	1.00	0.34	0.34
Sedentary behavior	1-class	16	−31,918.59	63,869.17	63,963.01	63,912.17	100				
	2-classes	14	−13,259.06	26,546.12	26,628.21	26,583.73	91,9	0.92	< 0.01	0.14	0.14
	3-classes	22	−13,113.08	26,250.16	26,320.54	26,282.41	90,2,8	0.93	< 0.01	0.01	0.01
	4-classes	30	−13,063.23	26,186.45	26,362.36	26,267.04	0,86,3,11	0.91	1.00	0.53	0.53

Notes: *LL* Log Likelihood; *AIC* Akaike information criteria; *BIC* Bayes Information criteria; *aBIC* sample-size adjusted BIC

**Fig. 1:**
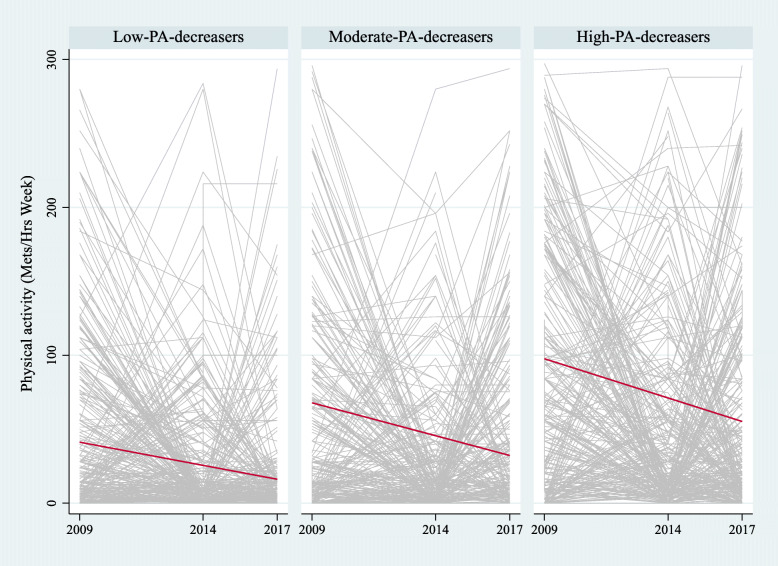
Trajectories of physical activity (PA) for the 3-class model. Notes: Low-PA-decreasers: Low baseline PA with decreasing trajectory (*n* = 1244, 39%). Moderate-PA-decreasers: Medium baseline PA with decreasing trajectory (*n* = 593, 18%). High-PA-decreasers: High baseline PA with decreasing trajectory (*n* = 1372, 43%)

**Fig. 2:**
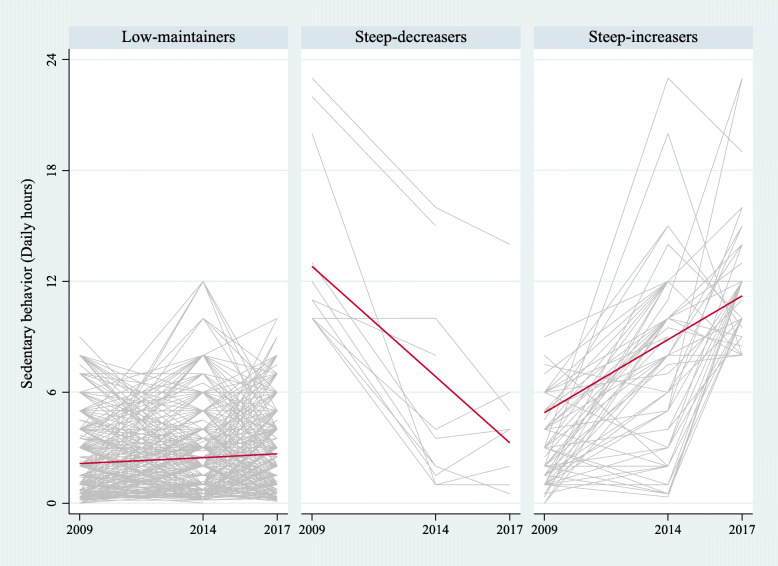
Trajectories of sedentary behavior (SB) for the 3-class model. Notes: Low-maintainers: Low baseline SB with stable trajectory (*n* = 2888, 90%). Steep-decreasers: High baseline SB with steep decreasing trajectory (*n* = 64, 2%). Steep-increasers: Low baseline SB with steep increasing trajectory (*n* = 257, 8%)

Figures 1 and 2 show the trajectories of PA and SB in the three-class model. For PA, the first class was identified as “low-PA-decreasers,” with the lowest baseline PA and the least steep decreasing trajectory. There were 1244 individuals in this class (39% of the sample) with an average baseline PA of 41.2 (SE = 0.5, *p*-value< 0.01) and a decline rate of − 3.1 (SE = 0.1, *p*-value< 0.01). The second class, “moderate-PA-decreasers,” had a medium baseline PA and a decreasing trajectory. This class had 593 individuals (18% of the sample) with moderate level of PA in baseline (intercept = 67.8, SE = 0.5, *p*-value< 0.01) and steeper decline (slope = − 4.4, SE = 0.1, *p*-value< 0.01). The third class, “high-PA-decreasers,” had a high baseline PA and a steep decreasing trajectory. This class had 1372 participants (43%) with a higher average baseline PA (intercept = 97.6, SE = 0.9, *p*-value< 0.01) but also with the steepest decreasing slope (− 5.3, SE = 0.1, *p*-value< 0.01) (Table 3).

**Table 3:** Parameter estimates for the trajectories of physical activity and sedentary behavior

	Physical activity			Sedentary behavior		
	Low-PA-decreasers	Moderate-PA-decreasers	High-PA-decreasers	Low-maintainers	Steep-decreasers	Steep-increasers
Sample size	*n* = 1244 (39%)	*n* = 593 (18%)	*n* = 1372 (43%)	*n* = 2888 (90%)	*n* = 64 (2%)	*n* = 257 (8%)
Average probability of class membership	0.99	1.00	0.99	0.98	0.92	0.87
Latent variable means						
Intercept	41.2 (SE = 0.5)	67.8 (SE = 0.5)	97.6 (SE = 0.9)	2.1 (SE = 0.1)	13.1 (SE = 0.6)	4.9 (SE = 0.3)
Slope	−3.1 (SE = 0.1)	−4.4 (SE = 0.1)	−5.3 (SE = 0.1)	0.1 (SE = 0.01)	−1.3 (SE = 0.1)	0.8 (SE = 0.1)
Latent variables variance						
Intercept variance	69.5 (SE = 5.4)	20.7 (SE = 4.7)	220.6 (SE = 15.5)	1.0 (SE = 0.1)	8.6 (SE = 2.5)	5.5 (SE = 1.1)
Residual variance	87.6 (SE = 3.6)	99.8 (5.4)	328.9 (SE = 11.8)	2.2 (SE = 0.2)	3.6 (0.3)	3.4 (SE = 0.5)

Note: *SE* standard error

For SB, we also identified three classes. The first group, “low-maintainers”, was composed of 2888 OA (90%), with the lowest baseline levels of SB (intercept = 2.1, SE = 0.1, *p*-value< 0.01) and a relatively stable trajectory (slope = 0.1, SE = 0.01, *p*-value< 0.01). The second group, “steep-decreasers”, had 64 individuals (2%), with the highest baseline SB (intercept = 13.1, SE = 0.6, *p*-value< 0.01) and a steep decreasing trajectory (slope = − 1.3, SE = 0.1, *p*-value< 0.01). Finally, the third group, “steep-increasers”, had 257 OA (8%), medium baseline SB levels (intercept = 4.9, SE = 0.3, *p*-value< 0.01), and a steeper increasing trajectory (slope = 0.8, SE = 0.1, *p*-value< 0.01) (Table 3).

Table 4 shows baseline health and sociodemographic characteristics by PA trajectories. In comparison to individuals in classes 1 (low baseline PA and less steep decreasing trajectory) and 2 (medium baseline PA and decreasing trajectory), older adults in class 3 (high baseline PA and steep decreasing trajectory), were younger (*p*-value< 0.01), mostly male (*p*-value< 0.01), had more years of formal education (*p*-value< 0.01) and better SES (*p*-value< 0.01). They also displayed a significantly lower prevalence of sarcopenia (*p*-value< 0.01) and multimorbidity (p-value< 0.01) and had the lowest levels of BMI (*p*-value< 0.01). Regarding SB trajectories, individuals with the worst trajectories (classes 2 and 3) had unfavorable health conditions than those in class 1. The former had a lower quality of life (*p* < 0.01), greater disability (*p* < 0.01), and a higher mortality rate (*p* < 0.01). Additionally, they had higher prevalences of multimorbidity (*p* < 0.01), frailty (*p* < 0.01), and sarcopenia (*p* < 0.01) (Additional file, Appendix 3).

**Table 4:** Baseline sociodemographic and health characteristics according to trajectories of physical activity

	Total	Low-PA-decreasers	Moderate-PA-decreasers	High-PA-decreasers	*p*-value
	*n* = 3209	*n* = 1244 (39%)	*n* = 593 (18%)	*n* = 1372 (43%)	
**Outcomes**					
Quality of life (mean, SD)	65.8 (14.1)	62.4 (14.7)	66.8 (13.4)	68.2 (13.1)	< 0.01
Disability (mean, SD)	16.6 (18.6)	27.3 (20.8)	13.3 (15.9)	9.3 (12.8)	< 0.01
All-cause mortality (%)	20.9	27.7	21.7	8.3	< 0.01
**Health and sociodemographics**					
Sex (female = 1) (%)	61.7	74.3	79.6	38.6	< 0.01
Age (mean, SD)	67.5 (10.3)	72.7 (8.3)	65.6 (7.8)	60.6 (9.1)	< 0.01
Years of formal education (mean, SD)	5.1 (4.4)	3.7 (3.7)	4.6 (4.4)	5.6 (4.5)	< 0.01
*Frailty (%)*					
Non-frail	44.8	53.8	44.4	36.2	
Prefrail	25.1	23.0	25.6	26.7	
Frail	30.1	23.2	30.0	37.1	< 0.01
Sarcopenia (%)	14.1	22.1	9.8	7.7	< 0.01
Body Mass Index (mean, SD -kg/m2)	28.3 (5.4)	28.5 (5.5)	28.8 (5.9)	27.8 (4.6)	< 0.01
Multimorbidity (%)	55.5	69.3	56.9	40.3	< 0.01
Health insurance (%)	71.1	72.8	71.0	71.0	0.72
Socioeconomic status (assets index) (mean, SD)	−0.03 (1.17)	−0.23 (1.12)	− 0.04 (1.16)	0.03 (1.18)	< 0.01

Notes: Cells are means (std. dev.) or percentages; p-value for ANOVA or chi-square tests; data for all-cause mortality refers to deaths reported in Wave 3

### Associations of PA and SB trajectories with QoL, disability, and all-cause mortality

The associations of PA and SB trajectories with the distal outcomes QoL, disability and all-cause mortality are depicted in Table 5. Regarding QoL, the classes with higher baseline PA levels (moderate-decreasers and high-decreasers) had the higher QoL levels (β = 2.90; 95% CI: 1.78;4.01; and β = 2.81; 95% CI: 1.64;3.98, respectively). Meanwhile, the group with the best SB trajectory (low-maintainers) also had the higher levels of QoL compared to the group of steep-increasers (β = − 3.70; 95% CI: − 5.31;-2.09).

**Table 5:** Estimated associations of physical activity and sedentary behavior trajectories with quality of life, disability, and all-cause mortality

	Quality of life		Disability		All-cause mortality	
	*Coefficient*	*95% CI*	*Coefficient*	*95% CI*	*Hazard ratio*	*95% CI*
**Physical activity trajectories**						
low-PA-decreasers	Ref.		Ref.		Ref.	
moderate-PA-decreasers	2.90	(1.78; 4.01)	−6.11	(−7.39; −4.82)	0.82	(0.63; 1.07)
high-PA-decreasers	2.81	(1.64; 3.98)	−7.60	(−8.95; −6.25)	0.33	(0.24; 0.44)
*Variance components*	*Estimate*	*95% CI*	*Estimate*	*95% CI*		
Subject	30.43	(21.99; 42.10)	71.58	(59.23; 86.51)		
Household	21.90	(14.41; 33.27)	12.26	(4.69; 32.04)		
*Intraclass correlation*						
Subject	0.33	(0.30; 0.36)	0.43	(0.40; 0.46)		
Household	0.14	(0.09; 0.20)	0.06	(0.02; 0.16)		
**Sedentary behavior** **trajectories**	*Coefficient*	*95% CI*	*Coefficient*	*95% CI*	*Hazard ratio*	*95% CI*
low-maintainers	Ref.		Ref.		Ref.	
steep-decreasers	−2.23	(−5.53; 1.08)	5.81	(1.94; 9.69)	0.40	(0.13; 1.27)
steep-increasers	−3.70	(−5.31; −2.09)	8.81	(6.92; 10.70)	1.44	(1.05; 1.96)
*Variance components*	*Estimate*	*95% CI*	*Estimate*	*95% CI*		
Subject	32.15	(23.26; 44.44)	81.95	(68.13; 90.58)		
Household	23.79	(15.72; 36.00)	11.31	(3.51; 36.43)		
*Intraclass correlation*						
Subject	0.35	(0.31; 0.38)	0.45	(0.42; 0.48)		
Household	0.15	(0.10; 0.22)	0.05	(0.02; 0.17)		

Notes: Linear regression model for quality of life and disability. Cox regression model for all-cause mortality. Results adjusted for covariates showed in Table 4 and the time of follow-up

The groups of moderate-PA-decreasers and high-PA-decreasers also had lower disability scores compared to the low-PA-decreasers group (β = − 6.11; 95% CI: − 7.39;-4.82; and β = − 7.60; 95% CI: − 8.95;-6.25, respectively). For SB, the steep-decreasers and steep-increasers groups showed worse levels of disability (β = 5.81; 95% CI: 1.94;9.69; and β = 8.81; 95% CI: 6.92;10.70, respectively) in comparison with the low-maintainers group.

For both outcomes (QoL and disability) and exposures (PA and SB) the results show that between-subject differences explain a greater proportion of the variance associated with the outcomes than between-household differences. Specifically, 33% of the variation in QoL is explained by individual differences (ICC = 0.33; 95% CI: 0.30–0.36) compared to 14% by household differences (ICC = 0.14; 95% CI: 0.09–0.20). Meanwhile, for disability, the observed data were 43% for between-subject differences (ICC = 0.43; 95% CI: 0.40–0.46), and 6% for between-household differences (ICC = 0.06; 95% CI: 0.02–0.16).

For all-cause mortality data, the median duration of follow-up was 2002 days (5.5 years), with an interquartile range of 1196 days. In total, 368 deaths were observed, equivalent to a mortality rate of 26.1 per 1000 person-years. Regarding the observed associations, the high-PA-decreasers group had a lower risk of dying than the low-PA-decreasers group (HR = 0.33; 95% CI: 0.24;0.44). Finally, the steep-increasers group in SB had a higher mortality risk than the low-maintainers group (HR = 1.44; 95% CI: 1.05;1.96).

## Discussion

In this study we identified different trajectories of PA and SB across eight years of follow-up in a national sample of older Mexican adults. Our findings are consistent with previous research indicating that aging is a heterogeneous process [33–35]. Three distinct longitudinal trajectories of PA and SB were found: low-PA-decreasers, moderate-PA-decreasers, and high-PA-decreasers for PA; and low-maintainers, steep-decreasers, and steep-increasers for SB.

All trajectories of PA had downward slopes of similar magnitude; however, they had different baseline levels. The low-PA-decreasers group had the lowest baseline PA; we consider it the most disadvantaged trajectory. Meanwhile, the moderate-PA-decreasers and high-PA-decreasers groups had higher PA baseline levels; despite their descending trajectories, they reached higher levels of PA than the low-decreasers group.

SB trajectories were more heterogeneous; one group had a relatively stable trajectory, another, a steep descending slope, and the last one, a steep upward slope. We consider the low-maintainers group as the best trajectory since they maintained low SB levels (2 h a day on average) throughout the follow-up. The steep-decreasers trajectory had significantly declining SB levels, but still started with very high baseline SB levels. The steep-increasers group probably holds the highest risk, given its steep SB increase throughout the study period.

Our study identified that typical trajectories of PA and SB do not exist, and therefore do not adequately describe a unique temporal trend of these variables among the older adult population. Similar results have been reported in previous studies. Sanchez-Sanchez et al, in one study with 1679 OA from Spain, reported five trajectories of PA using the Physical Activity Scale for the Elderly (PASE) during nine years of follow-up: high PA-consistent (HPAC); low PA-decreasing (LPAD); low PA-increasing (LPAI); moderate PA-consistent (MPAC); moderate PA-mildly decreasing (MPAMD) [9]. Using PASE, Laddu et al also reported three different trajectories of PA: high PA declining, moderate PA declining, and low PA declining [10].

Regarding the association between PA trajectories and mortality, Sanchez-Sanchez et al found that OA belonging to the LPAD trajectory had higher risk of all cause-mortality compared with those in HPAC (HR = 1.68 95%CI 1.21, 2.31) [9]. Laddu et al. found that OA in moderate PA-declining and high PA-declining groups had a lower risk of all-cause mortality, cardiovascular disease mortality, and non-cardiovascular disease mortality compared with OA in the low PA declining trajectory [10]. These associations agree with our findings, which identified that OA with low PA at baseline and a decreasing trend have a higher risk of mortality.

Sanchez-Sanchez et al also reported that OA in LPAD trajectory had higher probability of disability compared with those in HPAC (OR = 3.14 95% CI 1.59–6.19). Although they assessed disability with the Katz Index, their results are comparable with ours because OA in the moderate and high PA declining trajectories had lower levels of disability compared with the reference trajectory [9]. In our study, we used the WHODAS 2.0 to assess disability, which comprises more disability domains than just physical, and we still found that lower levels of PA can increase the levels of all types of disability.

Former studies have tested the association between PA with QoL. The study from Vallance et al found that women achieving the recommendations of PA (moderate-intensity PA for a minimum of 30 min on 5 d/wk. or vigorous-intensity activity for a minimum of 20 min on 3 d/wk., or combinations of moderate and vigorous intensity) had higher scores for the two components of QoL assessed with the RAND-12 Health Status Inventory (M_diff_ = 2.4, *P* = 0.008, *d* = 0.31 for physical component and, M_diff_ = 2.3, *P* = 0.011, *d* = 0.30 for mental component) compared with women who did not reach the recommendations [36]. Other studies have also analyzed this association and outlined how PA interventions could increase QoL [37, 38] However, after an extensive search, we did not find studies identifying PA trajectories and their association with QoL among community-dwelling OA.

SB has been widely studied because of its increasing prevalence in OA and its association with adverse health outcomes [5, 39]. A recent review of literature identified four prospective studies that evaluated the association between SB and mortality, reporting that increased SB is associated with an increased mortality [39]. Another systematic review analyzed two studies looking at the association between SB and QoL. The first did not find a significant association between SB and subjective well-being [40], while the second reported that SB is negatively associated with several domains of health-related QoL using the SF-36 instrument, such as physical functioning, physical role, body pain, vitality, social functioning, and mental health [41].

For the association between SB and disability, Dunlop et al reported that one additional hour of SB was associated with disability using the Katz scale (OR = 1.46 95%CI 1.07–1.98) [42]. The evidence seems to be consistent regarding the time spent in SB and adverse health outcomes in OA. Susanto et al identified five different trajectories of sitting time (hours/day) over a 12-year follow-up in Australian middle-aged women (>50y): low, medium, increasing, decreasing and high; the low pattern of sitting time per day was associated with reduced probability of frailty (OR = 0.86 95%CI 0.75, 0.98) [11]. To our knowledge, ours is the first study to look at SB trajectories in OA in relation to mortality, disability and QoL.

The causal pathways between PA, SB, and healthy aging have not been fully elucidated. PA seems to promote healthy aging by a combination of actions, rather than a single mechanism. These include: a beneficial change in inflammation biomarkers due to a reduction in adipose tissue, fat mass and consequently weight loss [43], immune system [44], modification of sex related hormones [45], upregulation of antioxidant defense system [46], and other aspects related with psychological modifications, like self-esteem as a contributor of functionality and QoL [47, 48]. Also, PA improves strength, aerobic capacity, balance, walking and flexibility, all of which are factors associated with reduction in falls, dependence, and disability [6].

Studies analyzing SB in animal models have found modifications in skeletal muscle regulatory genes of lipid metabolism [49]. Similar findings are reported in observational studies, identifying alterations in blood cholesterol [50], glucose, and free fatty acids [51, 52]. Also, SB has been associated with weight gain, increased waist circumference, and poor quality of diet [53]; these variations are related to cardiovascular disease and associated with mortality and poor physical performance [54]. Additionally, some psychological and sociological variables tied to QoL have been linked to SB, like loneliness, life satisfaction, and sense of belonging to a community [5].

### Strengths and limitations

Our study has some strengths. To the best of our knowledge, this is the first study conducted in LMIC to assess the longitudinal trajectories of PA and SB, and their associations with QoL, disability, and all-cause mortality among OA population. Additionally, we used several outcomes that could be highly significant for this population group. There were also some limitations to our study. First, we used self-reported PA and SB data instead of objective measurements, which could have led to an overestimation of PA and underestimation of SB. In particular, the reported time of SB (two hours on average) seems low given that some studies have reported higher levels of SB among older adults [55]. However, previous studies with cross-sectional data (including the six countries in the SAGE study) have reported similar levels in the average number of SB daily hours [56, 57]. Additionally, it is important to note that our SB measurement is based on a single question investigating the time that older adults spend in a sitting position (excluding sleep time). Other SB measurements are based on complete batteries/questionnaires that include different activities not considered in the SAGE study [58, 59]. This bias could cause some older adults to be miscategorized in some of the estimated trajectories. Second, recall and survivor bias can affect the results of epidemiological studies with older adults. It is possible that participants in our study with worse disability and QoL died at younger ages. Therefore, the healthiest individuals would be interviewed, and the associations may have been underestimated. Third, losses to follow-up between waves (15% after Wave 1 and 17% after Wave 2) seem high. Although, the SAGE study has added new individuals in each round to compensate for these losses. Still, when comparing the excluded individuals with those in the analytical sample, the former were older and less healthy. Consequently, the observed associations could be underestimated since younger and healthier individuals tend to have a better quality of life, less disability, and lower mortality rates.

## Conclusions

Our key findings are that PA and SB have heterogeneous trajectories, and that QoL, disability, and mortality are consistently related to the worse trajectories. These findings imply that, even in the same age cohort, populations of OA with lower levels of PA and higher time spent in SB should be prioritized to prevent mortality, disability, and worsening QoL. Our results also highlight the need for health policies and prevention strategies in middle-aged adults to promote PA and minimize SB. Further studies should consider these activities/behaviors as exposures that vary throughout life to identify vulnerable groups who would benefit the most from physical activation interventions. This is particularly important in LMIC, like Mexico, in order to maximize resources and population health.

## Supplementary Information

**Additional file 1.**

## Data Availability

The datasets supporting the conclusions of this article are available in the WHO repository, http://apps.who.int/healthinfo/systems/surveydata/index.php/catalog/sage/about.

## Abbreviations

OA: Older adults
PA: Physical activity
LMIC: Low and middle income countries
SB: Sedentary behaviors
QoL: Quality of life
WHO: World Health Organization
SAGE: Study on global AGEing and adult health
WHOQOL: WHO Quality of Life
WHODAS: WHO Disability Assessment Schedule
GPAQ: Global Physical Activity Questionnaire
MET: Metabolic equivalent task
SES: Socioeconomic status
BMI: Body mass index
GMM: Growth mixture model
BIC: Bayesian information criteria
AIC: Akaike information criteria
aBIC: Adjusted Bayesian information criteria
LRT: Likelihood ratio test
FML: Full maximum likelihood
EM: Expectation-maximization
HR: Hazard ratio
CI: Confidence interval
SE: Standard error
PASE: Physical Activity Scale for the Elderly
HPAC: High PA-consistent
LPAD: Low PA-decreasing
LPAI: Low PA-increasing
MPAC: Moderate PA-consistent
MPAMD: Moderate PA-mildly decreasing
M_diff_: Mean difference
