# Supplementary material for: Physical activity and sedentary behavior trajectories and their associations with quality of life, disability, and all-cause mortality
Source: Eur Rev Aging Phys Act. 2022 Apr 29;19:13. doi: 10.1186/s11556-022-00291-3 (PMC9052456; doi:10.1186/s11556-022-00291-3)
Supplement: Supplementary file 1 — Additional file 1. [file 11556_2022_291_MOESM1_ESM.docx]

**Appendix 1**

Figure S1. Flow diagram for the analytical sample

Baseline sample (Wave 1-2009)

N=2,404

Excluded from follow-up (Wave 2)

n=642

273 Dead

369 Lost to follow-up

Wave 2-2014

N= 2380

Refreshed sample=618

Excluded from follow-up (Wave 3)

n=672

368 Dead

304 Lost to follow-up

Wave 3-2017

N=2318

Refreshed sample=610

Total sample

N=3277

Excluded from analytical sample: missing values on some covariates

n=68

Analytical sample

N=3209

With 3 measurements=884

With 2 measurements=2325

**Appendix 2**

MPLUS syntax code

**Growth Mixture Modeling with class-specific random intercepts**

**Title: Physical activity trajectories**

**Data:** file = data.dat;

**Variable:** names are id pa1 pa2 pa3;

Missing are all (-9999);

usevar pa1 pa2 pa3;

IDVARIABLE=id;

classes=c(3); *!VALUES RANGED FROM 1 TO 4*

**Analysis:** type=mixture;

SETSEED=132336

LRTBOOTSTRAP = 30;

starts=750 50;

LRTSTARTS = 40 40 200 100;

STITERATIONS=20;

estimator=MLR;

**Model:** %overall%

i s | pa1@0 pa2@1 pa3@2;

s@0;

*!VALUES RANGED FROM 1 TO 4*

%c#1%

[i-s];

i;

%c#2%

[i-s];

i;

%c#3%

[i-s];

i;

**Output:** TECH7 TECH11 TECH14;

**Savedata:** FILE IS pa.txt;

save=cprobabilities;

**Title: Sedentary behavior trajectories**

**Data:** file = data.dat;

**Variable:** names are id sb1 sb2 sb3;

Missing are all (-9999);

usevar sb1 sb2 sb3;

IDVARIABLE=id;

classes=c(3); *!VALUES RANGED FROM 1 TO 4*

**Analysis:** type=mixture;

SETSEED=132330

LRTBOOTSTRAP = 30;

starts=750 50;

LRTSTARTS = 40 40 200 100;

STITERATIONS=20;

estimator=MLR;

**Model:** %overall%

i s | sb1@0 sb2@1 sb3@2;

s@0;

*!VALUES RANGED FROM 1 TO 4*

%c#1%

[i-s];

i;

%c#2%

[i-s];

i;

%c#3%

[i-s];

i;

**Output:** TECH7 TECH11 TECH14;

**Savedata:** FILE IS sb.txt;

save=cprobabilities;

**Appendix 3**

Table S1. Baseline sociodemographic and health characteristics according to trajectories of sedentary behavior

|  | Total | Low-maintainers | Steep-decreasers | Steep-increasers | p-value |
| --- | --- | --- | --- | --- | --- |
|  | n=3209 | n=2888 (90%) | n=64 (2%) | n=257 (8%) |  |
| **Outcomes** |  |  |  |  |  |
| Quality of life (mean, SD) | 65.8 (14.1) | 66.3 (13.6) | 60.3 (16.9) | 59.1 (16.4) | <0.01 |
| Disability (mean, SD) | 16.6 (18.6) | 15.4 (16.9) | 25.8 (26.1) | 33.1 (25.4) | <0.01 |
| All-cause mortality (%) | 20.9 | 7.0 | 3.0 | 28.4 | <0.01 |
| **Health and sociodemographics** |  |  |  |  |  |
| Sex (female=1) (%) | 61.7 | 62.6 | 60.6 | 59.0 | 0.63 |
| Age (mean, SD) | 67.5 (10.3) | 66.0 (9.5) | 69.1 (13.8) | 72.4 (9.9) | <0.01 |
| Years of formal education (mean, SD) | 5.1 (4.4) | 4.9 (4.3) | 6.5 (5.5) | 3.9 (3.8) | <0.01 |
| *Frailty (%)* |  |  |  |  |  |
| Non-frail | 44.8 | 54.9 | 57.6 | 32.6 |  |
| Prefrail | 25.1 | 37.4 | 21.2 | 13.7 |  |
| Frail | 30.1 | 7.7 | 31.2 | 34.0 | <0.01 |
| Sarcopenia (%) | 14.1 | 12.6 | 16.0 | 22.6 | <0.01 |
| Body Mass Index (mean, SD -kg/m2) | 28.3 (5.4) | 28.3 (5.3) | 29.3 (5.3) | 29.5 (6.2) | 0.02 |
| Multimorbidity (%) | 55.5 | 55.2 | 65.5 | 71.7 | <0.01 |
| Health insurance (%) | 71.1 | 71.6 | 65.2 | 72.9 | 0.75 |
| Socioeconomic status (assets index) (mean, SD) | -0.03 (1.17) | -0.08 (1.16) | 0.11 (1.24) | -0.11 (1.11) | 0.62 |

Notes: Cells are means (std. dev.) or percentages; p-value for ANOVA or chi-square tests; data for all-cause mortality refers to deaths reported in Wave 3

**Appendix 4**

Table S2. Estimated associations of physical activity trajectories with quality of life, disability, and all-cause mortality

|  | **Quality of life** | | | | | |
| --- | --- | --- | --- | --- | --- | --- |
|  | Model 1 | | Model 2 | | Model 3 | |
|  | Coefficient | 95% CI | Coefficient | 95% CI | Coefficient | 95% CI |
| **Physical activity trajectories** |  |  |  |  |  |  |
| low-PA-decreasers | Ref. |  | Ref. |  | Ref. |  |
| moderate-PA-decreasers | 4.37 | (3.17 ; 5.57) | 2.95 | (1.83 ; 4.07) | 2.90 | (1.78 ; 4.01) |
| high-PA-decreasers | 3.98 | (2.77 ; 5.19) | 2.92 | (1.74 ; 4.10) | 2.81 | (1.64 ; 3.98) |
| Sex (female=1) | -1.64 | (-2.56 ; -0.72) | -0.41 | (-1.29 ; 0.48) | -0.71 | (-1.60 ; 0.17) |
| Age | -0.02 | (-0.07 ; 0.04) | 0.20 | (0.15 ; 0.26) | 0.22 | (0.17 ; 0.27) |
| Years of formal education |  |  | 0.37 | (0.27 ; 0.46) | 0.39 | (0.30 ; 0.49) |
| *Frailty* |  |  |  |  |  |  |
| Non-frail |  |  | Ref. |  | Ref. |  |
| Prefrail |  |  | -2.93 | (-3.65 ; -2.22) | -2.87 | (-3.58 ; -2.15) |
| Frail |  |  | -8.73 | (-9.89 ; -7.58) | -8.29 | (-9.46 ; -7.11) |
| Sarcopenia |  |  | -0.65 | (-1.65 ; 0.34) | -0.73 | (-1.73 ; 0.28) |
| Body Mass Index (kg/m2) |  |  | -0.05 | (-0.13 ; 0.02) | -0.03 | (-0.11 ; 0.04) |
| Multimorbidity |  |  | -3.65 | (-4.44 ; -2.87) | -3.64 | (-4.43 ; -2.86) |
| Health insurance |  |  | 1.05 | (0.07 ; 2.02) | 1.09 | (0.11 ; 2.07) |
| Socioeconomic status (assets index) | |  | 1.78 | (1.45 ; 2.11) | 1.83 | (1.50 ; 2.16) |
| Sedentary behavior (daily hours) |  |  |  |  | -0.44 | (-0.58 ; -0.29) |

|  | **Disability** | | | | | |
| --- | --- | --- | --- | --- | --- | --- |
|  | Model 1 | | Model 2 | | Model 3 | |
|  | Coefficient | 95% CI | Coefficient | 95% CI | Coefficient | 95% CI |
| **Physical activity trajectories** |  |  |  |  |  |  |
| low-PA-decreasers | Ref. |  | Ref. |  | Ref. |  |
| moderate-PA-decreasers | -9.12 | (-10.64 ; -7.61) | -6.23 | (-7.55 ; -4.91) | -6.11 | (-7.39 ; -4.82) |
| high-PA-decreasers | -12.31 | (-13.83 ; -10.78) | -7.86 | (-9.25 ; -6.47) | -7.60 | (-8.95 ; -6.25) |
| Sex (female=1) | 1.37 | (0.18 ; 2.56) | 0.27 | (-0.79 ; 1.32) | 0.92 | (-0.11 ; 1.96) |
| Age | 0.36 | (0.30 ; 0.42) | 0.21 | (0.15 ; 0.28) | 0.17 | (0.11 ; 0.23) |
| Years of formal education |  |  | -0.24 | (-0.35 ; -0.14) | -0.31 | (-0.41 ; -0.20) |
| *Frailty* |  |  |  |  |  |  |
| Non-frail |  |  | Ref. |  | Ref. |  |
| Prefrail |  |  | 4.11 | (3.34 ; 4.88) | 4.02 | (3.25 ; 4.78) |
| Frail |  |  | 15.95 | (14.68 ; 17.21) | 14.71 | (13.44 ; 15.98) |
| Sarcopenia |  |  | 1.09 | (-0.02 ; 2.20) | 1.14 | (0.04 ; 2.24) |
| Body Mass Index (kg/m2) |  |  | 0.24 | (0.16 ; 0.32) | 0.19 | (0.11 ; 0.27) |
| Multimorbidity |  |  | 2.93 | (2.04 ; 3.81) | 2.92 | (2.05 ; 3.79) |
| Health insurance |  |  | -1.09 | (-2.16 ; -0.03) | -1.13 | (-2.19 ; -0.08) |
| Socioeconomic status (assets index) | |  | -0.60 | (-0.97 ; -0.24) | -0.76 | (-1.12 ; -0.40) |
| Sedentary behavior (daily hours) |  |  |  |  | 1.14 | (0.99 ; 1.30) |

|  | **All-cause mortality** | | | | | |
| --- | --- | --- | --- | --- | --- | --- |
|  | Model 1 | | Model 2 | | Model 3 | |
|  | Hazard ratio | 95% CI | Hazard ratio | 95% CI | Hazard ratio | 95% CI |
| **Physical activity trajectories** |  |  |  |  |  |  |
| low-PA-decreasers | Ref. |  | Ref. |  | Ref. |  |
| moderate-PA-decreasers | 0.67 | (0.54 ; 0.82) | 0.80 | (0.62 ; 1.05) | 0.82 | (0.63 ; 1.07) |
| high-PA-decreasers | 0.23 | (0.19 ; 0.29) | 0.30 | (0.23 ; 0.41) | 0.33 | (0.24 ; 0.44) |
| Sex (female=1) | 0.49 | (0.41 ; 0.59) | 0.46 | (0.37 ; 0.57) | 0.50 | (0.40 ; 0.62) |
| Years of formal education |  |  | 0.98 | (0.95 ; 1.01) | 0.98 | (0.95 ; 1.01) |
| *Frailty* |  |  |  |  |  |  |
| Non-frail |  |  | Ref. |  | Ref. |  |
| Prefrail |  |  | 1.40 | (1.08 ; 1.80) | 1.37 | (1.06 ; 1.76) |
| Frail |  |  | 2.04 | (1.46 ; 2.84) | 1.82 | (1.30 ; 2.55) |
| Sarcopenia |  |  | 1.49 | (1.16 ; 1.91) | 1.48 | (1.16 ; 1.91) |
| Body Mass Index (kg/m2) |  |  | 0.97 | (0.95 ; 0.99) | 0.97 | (0.95 ; 0.99) |
| Multimorbidity |  |  | 1.33 | (1.07 ; 1.68) | 1.36 | (1.09 ; 1.71) |
| Health insurance |  |  | 0.97 | (0.77 ; 1.23) | 0.98 | (0.78 ; 1.25) |
| Socioeconomic status (assets index) | |  | 1.04 | (0.95 ; 1.15) | 1.05 | (0.95 ; 1.16) |
| Sedentary behavior (daily hours) |  |  |  |  | 1.06 | (1.03 ; 1.09) |

Table S3. Estimated associations of sedentary behavior trajectories with quality of life, disability, and all-cause mortality

|  | **Quality of life** | | | | | |
| --- | --- | --- | --- | --- | --- | --- |
|  | Model 1 | | Model 2 | | Model 3 | |
|  | Coefficient | 95% CI | Coefficient | 95% CI | Coefficient | 95% CI |
| **Sedentary behavior trajectories** |  |  |  |  |  |  |
| low-maintainers | Ref. |  | Ref. |  | Ref. |  |
| steep-decreasers | -2.67 | (-6.03 ; 0.68) | -2.32 | (-5.63 ; 0.99) | -2.23 | (-5.53 ; 1.08) |
| steep-increasers | -8.27 | (2.77 ; 5.19) | -3.91 | (-5.52 ; -2.30) | -3.70 | (-5.31 ; -2.09) |
| Sex (female=1) | -2.69 | (-3.49 ; -1.88) | -1.22 | (-2.00 ; -0.44) | -1.09 | (-1.88 ; -0.30) |
| Age | -0.04 | (-0.08 ; 0.01) | 0.15 | (0.11 ; 0.20) | 0.16 | (0.11 ; 0.21) |
| Years of formal education |  |  | 0.38 | (0.28 ; 0.47) | 0.38 | (0.29 ; 0.48) |
| *Frailty* |  |  |  |  |  |  |
| Non-frail |  |  | Ref. |  | Ref. |  |
| Prefrail |  |  | -3.02 | (-3.74 ; -2.30) | -2.93 | (-3.65 ; -2.21) |
| Frail |  |  | -8.72 | (-9.89 ; -7.54) | -8.58 | (-9.76 ; -7.40) |
| Sarcopenia |  |  | -0.88 | (-1.89 ; 0.14) | -0.89 | (-1.90 ; 0.12) |
| Body Mass Index (kg/m2) |  |  | -0.04 | (-0.12 ; 0.03) | -0.04 | (-0.11 ; 0.03) |
| Multimorbidity |  |  | -3.84 | (-4.63 ; -3.05) | -3.82 | (-4.61 ; -3.03) |
| Health insurance |  |  | 1.14 | (0.16 ; 2.13) | 1.16 | (0.18 ; 2.15) |
| Socioeconomic status (assets index) | |  | 1.80 | (1.47 ; 2.14) | 1.82 | (1.49 ; 2.15) |
| Physical activity (METS/hours per week) |  |  |  |  | 0.05 | (0.01 ; 0.09) |

|  | **Disability** | | | | | |
| --- | --- | --- | --- | --- | --- | --- |
|  | Model 1 | | Model 2 | | Model 3 | |
|  | Coefficient | 95% CI | Coefficient | 95% CI | Coefficient | 95% CI |
| **Sedentary behavior trajectories** |  |  |  |  |  |  |
| low-maintainers | Ref. |  | Ref. |  | Ref. |  |
| steep-decreasers | 9.75 | (5.59 ; 13.91) | 5.91 | (2.03 ; 9.79) | 5.81 | (1.94 ; 9.69) |
| steep-increasers | 18.62 | (16.76 ; 20.48) | 8.85 | (6.96 ; 10.73) | 8.81 | (6.92 ; 10.70) |
| Sex (female=1) | 5.27 | (4.23 ; 6.30) | 2.62 | (1.68 ; 3.56) | 2.50 | (1.56 ; 3.45) |
| Age | 0.60 | (0.55 ; 0.66) | 0.37 | (0.32 ; 0.43) | 0.36 | (0.31 ; 0.42) |
| Years of formal education |  |  | -0.27 | (-0.38 ; -0.16) | -0.27 | (-0.38 ; -0.16) |
| *Frailty* |  |  |  |  |  |  |
| Non-frail |  |  | Ref. |  | Ref. |  |
| Prefrail |  |  | 4.27 | (3.34 ; 4.88) | 4.22 | (3.44 ; 4.99) |
| Frail |  |  | 16.03 | (14.75 ; 17.33) | 15.92 | (14.62 ; 17.21) |
| Sarcopenia |  |  | 1.34 | (0.21 ; 2.47) | 1.35 | (0.22 ; 2.49) |
| Body Mass Index (kg/m2) |  |  | 0.24 | (0.16 ; 0.33) | 0.24 | (0.16 ; 0.33) |
| Multimorbidity |  |  | 3.32 | (2.42 ; 4.21) | 3.33 | (2.44 ; 4.23) |
| Health insurance |  |  | -1.20 | (-2.28 ; -0.12) | -1.19 | (-2.27 ; -0.11) |
| Socioeconomic status (assets index) | |  | -0.75 | (-1.12 ; -0.38) | -0.75 | (-1.12 ; -0.38) |
| Physical activity (METS/hours per week) |  |  |  |  | -0.05 | (-0.09 ; -0.01) |

|  | **All-cause mortality** | | | | | |
| --- | --- | --- | --- | --- | --- | --- |
|  | Model 1 | | Model 2 | | Model 3 | |
|  | Hazard ratio | 95% CI | Hazard ratio | 95% CI | Hazard ratio | 95% CI |
| **Sedentary behavior trajectories** |  |  |  |  |  |  |
| low-maintainers | Ref. |  | Ref. |  | Ref. |  |
| steep-decreasers | 0.55 | (0.23 ; 1.33) | 0.45 | (0.14 ; 1.40) | 0.40 | (0.13 ; 1.27) |
| steep-increasers | 2.29 | (1.85 ; 2.84) | 1.60 | (1.18 ; 2.18) | 1.44 | (1.05 ; 1.96) |
| Sex (female=1) | 0.82 | (0.70 ; 0.96) | 0.69 | (0.56 ; 0.84) | 0.63 | (0.51 ; 0.77) |
| Years of formal education |  |  | 0.96 | (0.94 ; 0.99) | 0.96 | (0.93 ; 0.99) |
| *Frailty* |  |  |  |  |  |  |
| Non-frail |  |  | Ref. |  | Ref. |  |
| Prefrail |  |  | 1.50 | (1.17 ; 1.93) | 1.40 | (1.09 ; 1.80) |
| Frail |  |  | 2.60 | (1.88 ; 3.60) | 2.22 | (1.60 ; 3.11) |
| Sarcopenia |  |  | 1.87 | (1.46 ; 2.39) | 1.81 | (1.41 ; 2.31) |
| Body Mass Index (kg/m2) |  |  | 0.96 | (0.94 ; 0.98) | 0.96 | (0.94 ; 0.98) |
| Multimorbidity |  |  | 1.58 | (1.27 ; 1.96) | 1.58 | (1.26 ; 1.96) |
| Health insurance |  |  | 0.99 | (0.78 ; 1.26) | 0.97 | (0.76 ; 1.23) |
| Socioeconomic status (assets index) | |  | 1.03 | (0.93 ; 1.14) | 1.02 | (0.93 ; 1.14) |
| Physical activity (METS/hours per week) |  |  |  |  | 0.99 | (0.99 ; 0.99) |

**Appendix 5**

**Table S4.** STROBE Statement—Checklist of items that should be included in reports of *cohort studies*

|  | Item No | Recommendation | Page number |
| --- | --- | --- | --- |
| **Title and abstract** | 1 | (*a*) Indicate the study’s design with a commonly used term in the title or the abstract | 1 |
|  |  | (*b*) Provide in the abstract an informative and balanced summary of what was done and what was found | 1, 2 |
| Introduction | | |  |
| Background/rationale | 2 | Explain the scientific background and rationale for the investigation being reported | 3-4 |
| Objectives | 3 | State specific objectives, including any prespecified hypotheses | 4 |
| Methods | | |  |
| Study design | 4 | Present key elements of study design early in the paper | 4 |
| Setting | 5 | Describe the setting, locations, and relevant dates, including periods of recruitment, exposure, follow-up, and data collection | 4-5 |
| Participants | 6 | (*a*) Give the eligibility criteria, and the sources and methods of selection of participants. Describe methods of follow-up | 5 |
|  |  | (*b*) For matched studies, give matching criteria and number of exposed and unexposed | N/A |
| Variables | 7 | Clearly define all outcomes, exposures, predictors, potential confounders, and effect modifiers. Give diagnostic criteria, if applicable | 5-7 |
| Data sources/ measurement | 8* | For each variable of interest, give sources of data and details of methods of assessment (measurement). Describe comparability of assessment methods if there is more than one group | 5-7 |
| Bias | 9 | Describe any efforts to address potential sources of bias | 7 |
| Study size | 10 | Explain how the study size was arrived at | 4-5 |
| Quantitative variables | 11 | Explain how quantitative variables were handled in the analyses. If applicable, describe which groupings were chosen and why | 5-7 |
| Statistical methods | 12 | (*a*) Describe all statistical methods, including those used to control for confounding | 7-8 |
|  |  | (*b*) Describe any methods used to examine subgroups and interactions | 7-9 |
|  |  | (*c*) Explain how missing data were addressed | N/A |
|  |  | (*d*) If applicable, explain how loss to follow-up was addressed | N/A |
|  |  | (*e*) Describe any sensitivity analyses | N/A |
| Results | | |  |
| Participants | 13* | (a) Report numbers of individuals at each stage of study—eg numbers potentially eligible, examined for eligibility, confirmed eligible, included in the study, completing follow-up, and analysed | Figure S1 |
|  |  | (b) Give reasons for non-participation at each stage | Figure S1 |
|  |  | (c) Consider use of a flow diagram | Figure S1 |
| Descriptive data | 14* | (a) Give characteristics of study participants (eg demographic, clinical, social) and information on exposures and potential confounders | 9, 10 |
|  |  | (b) Indicate number of participants with missing data for each variable of interest | Figure S1 |
|  |  | (c) Summarise follow-up time (eg, average and total amount) | Figure S1 |
| Outcome data | 15* | Report numbers of outcome events or summary measures over time | 13-14 |
| Main results | 16 | (*a*) Give unadjusted estimates and, if applicable, confounder-adjusted estimates and their precision (eg, 95% confidence interval). Make clear which confounders were adjusted for and why they were included | 10-13 |
|  |  | (*b*) Report category boundaries when continuous variables were categorized | N/A |
|  |  | (*c*) If relevant, consider translating estimates of relative risk into absolute risk for a meaningful time period | N/A |
| Other analyses | 17 | Report other analyses done—eg analyses of subgroups and interactions, and sensitivity analyses | N/A |
| Discussion | | |  |
| Key results | 18 | Summarise key results with reference to study objectives | 14-15 |
| Limitations | 19 | Discuss limitations of the study, taking into account sources of potential bias or imprecision. Discuss both direction and magnitude of any potential bias | 17 |
| Interpretation | 20 | Give a cautious overall interpretation of results considering objectives, limitations, multiplicity of analyses, results from similar studies, and other relevant evidence | 14-16 |
| Generalisability | 21 | Discuss the generalisability (external validity) of the study results | 17 |
| Other information | | |  |
| Funding | 22 | Give the source of funding and the role of the funders for the present study and, if applicable, for the original study on which the present article is based | 19 |
